# Neurodegeneration correlates of iron-related lesions and leptomeningeal inflammation in multiple sclerosis clinical subtypes

**DOI:** 10.1007/s00234-025-03595-0

**Published:** 2025-03-25

**Authors:** Aigli G Vakrakou, Ioannis Papadopoulos, Maria-Evgenia Brinia, Dimitrios Karathanasis, Dimitrios Panaretos, Panos Stathopoulos, Anastasia Alexaki, Varvara Pantoleon, Efstratios Karavasilis, Georgios Velonakis, Leonidas Stefanis, Maria-Eleftheria Evangelopoulos, Constantinos Kilidireas

**Affiliations:** 1 Neuroimmunology Unit,1st Department of Neurology, School of Medicine, Aiginition Hospital, National and Kapodistrian University of Athens, NKUA, Athens, Greece; 2 Multiple Sclerosis and Demyelinating Diseases Unit, Center of Expertise for Rare Demyelinating and Autoimmune Diseases of CNS, First Department of Neurology, School of Medicine, National and Kapodistrian University of Athens, NKUA, Aiginition University Hospital, Athens, Greece; 3 Research Unit of Radiology,2nd Department of Radiology, Medical School, National and Kapodistrian University of Athens, Athens, Greece; 4 Department of Statistics and Insurance Science, School of Economic Sciences, University of Western, Kozani, Macedonia; 5 Laboratory of Medical Physics, School of Medicine, Democritus University of Thrace, Alexandroupolis, 68100 Greece; 6 Department of Neurology, Henry Dunant Hospital Center, Athens, Greece

**Keywords:** Multiple sclerosis, Paramagnetic rim-lesions, Susceptibility-susceptibility images, EDSS, Leptomeningeal inflammation, Thalamus, Cortical thickness

## Abstract

**Purpose:**

The aim of this study was to investigate the significant implications of different types of lesions as assessed by QSM (quantitative-susceptibility-mapping) as well as leptomeningeal contrast-enhancement in a cohort of Relapsing-Remitting (RR) and Primary Progressive (PP) MS patients and to assess their association with clinical disability and MRI-measures of brain structural damage.

**Methods:**

Different types of white-matter lesions were identified and quantified using QSM in 24 RRMS and 15 PPMS (11 patients with follow-up MRI). Leptomeningeal contrast-enhancement (LMCE; foci) was assessed on 3D-FLAIR post-gadolinium.

**Results:**

Both RRMS and PPMS presented PRL (paramagnetic-rim lesions) and LMCE, with PPMS showing a trend towards more LMCE (RRMS 37%, PPMS 53%). In QSM RRMS patients showed more hyperintense white-matter lesions with greater lesion volume. In RRMS PRL correlated with disease duration and lesion burden especially the volume of juxtacortical Flair-hyperintense lesions. Besides, the presence of PRL lesions in PPMS was associated with subcortical atrophy mainly thalamus and pallidum volumetry. In all MS-cohort, patients with more than 3-PRLs exhibited reduced regional cortical thickness in specific temporal areas and post/para central gyrus. Forest-analysis selected age, increased NAWM (normal appearing white-matter) QSM intensity, total lesion volume and the presence of LMCE as informative predictors of cortical thickness. After anti-CD20 treatment, no significant change was observed regarding the number of PRL and LMCE, but the percentage of PRL lesions over the total lesion types and the QSM rim intensity increased.

**Conclusion:**

Our findings suggest that QSM-lesion types and leptomeningeal inflammation capture different aspects of progressive disease biology in both RRMS and PPMS.

**Supplementary Information:**

The online version contains supplementary material available at 10.1007/s00234-025-03595-0.

## Introduction

Multiple sclerosis (MS) is a chronic disabling neurological condition affecting the CNS, which most commonly becomes clinically evident during early adult life. According to neuropathology studies mainly from post-mortem brain tissue, MS lesions can be classified as active, mixed active–inactive, inactive and remyelinated depending on the features on the existence or absence of ongoing demyelination and the characteristics of microglia and macrophages within the lesion [1]. Active lesions are characterized by massive infiltration of macrophages mostly recruited from the periphery, and active phagocytosis of myelin products. Mostly T cells (CD8 T cells) and less B cells infiltrate the lesions and are centered around veins and venules. Mixed active/inactive lesions display an hypocellular lesion center along with a rim of activated macrophages/microglial cells where active demyelination occurs. Inactive lesions are sharply demarcated from the NAWM (normal appearing white matter) and are hypocellular with no active demyelination but evident axonal loss [2]. Active lesions are mainly observed in relapsing disease forms whereas mixed and inactive in more progressive disease forms [3]. Advanced MRI (magnetic resonance imaging) techniques have provided substantial help in phenotyping of brain lesions in patients, thus providing clinicians with the opportunity to capture aspects of active and/or compartmentalized inflammation during disease course. Neuropathology hallmark of chronic active lesions is the accumulation of iron in macrophages/microglial cells in the lesion edges, where demyelination still occurs even if the blood-brain barrier (BBB) is closed.

Quantitative susceptibility mapping (QSM) and post-mortem investigations have revealed that the accumulation of iron along lesion edges manifests as a rim-shaped signal surrounding chronic white matter (WM) lesions (PRL; paramagnetic lesions) [4, 5]. Iron rims are more destructive (damage in myelin and axons is higher in rim-lesions), signify persistent inflammation, expand over time and block repair mechanisms [5–7]. QSM could be used to discriminate other types of lesions than chronic active with less sensitivity [8]. The combination of QSM along with other quantitative MRI techniques have recently been useful in capturing different types of lesions in vivo [9].

Perturbation of the meningeal blood-brain barrier, meningeal inflammation with B cell follicle accumulation and/or alterations in the glymphatic/lymphatic system are considered pathomechanisms accounting for meningeal enhancement in MS [10]. In post-mortem investigations and ex-vivo biopsy tissue studies, meningeal inflammation has been spatially linked to subpial cortical demyelination and cortical atrophy [11, 12]. Post-contrast FLAIR sequences have described leptomeningeal contrast-enhancement (LMCE; foci) in MS, especially in the progressive forms [13]. High resolution FLAIR MRI with a delay after contrast administration has been shown to improve sensitivity for the detection of low concentrations of contrast in CSF (cerebrospinal fluid) [14–18]. However, there is no solid evidence regarding the pathological correlation of LMCE with ectopic germinal centers. It is generally considered LMCE to represent areas of meningeal inflammation that could lead to blood meningeal barrier perturbation [19–21].

The goal of this study was to assess for the first time the association between different types of QSM lesions (including PRL) and the presence of LMCE with clinical disability, global measures of structural tissue damage, gray matter atrophy (cortical and subcortical), global and regional cortical thickness as well as their interactions in a cohort of remitting (RR) MS and PPMS patients. LMCE and PRLs may both be indicative of chronic and diffuse inflammatory changes in MS that may be restricted to the CNS [10, 20, 22]. Thus, it is assumed that both may be co-linked biomarkers of compartmentalized inflammation. Therefore, we evaluated the relationship between PRLs and LCME on 3T MRI in MS patients to further explore their potential link.

## Materials and methods

We conducted a prospective MRI study including patients with MS: 24 RRMS and 15 PPMS patients diagnosed according to the 2017 revised McDonald criteria [23]. All patients were in remission, without clinical relapses or significant disease deterioration at the time point of initial MRI assessment.

Subjects’ demographics, binding genotype, MRI and clinical data are reported in Table 1. This was a prospective study composed of 39 patients, 17 females and 22 males with a mean of age 41.31 years (mean ± 12,42 SD), disease duration of 64.38 ± 56.92 months, and a median EDSS (Expanded Disability Status Scale) of 2.5. Regarding disease subtypes, we included 24 RRMS and 15 PPMS patients with a mean age of 38 and 47, respectively (Table 1). Glatiramer acetate, fingolimod, mycophenolate mofetil, azathioprine and anti-CD20 antibodies (Rituximab, Ocrelizumab and Ofatumumab) were among the prophylactic treatments administered to our cohort (Table 1). At the time of the MRI imaging, eight PPMS patients and seven RRMS patients were drug naïve.

**Table 1:** Main clinical, serological, and radiological characteristics of the studied cohort

	RRMS	PPMS	*p*-values	
**Demographic and Clinical data**				
	Mean (± SD)	Mean (± SD)		
Sex (female: male)	11:13	6:9	0.12	
Age at disease diagnosis (years)	32 (13.21)	41.33(10.31)	0.02	
Age (years)	37.92 (11.77)	46.73 (11.83)	0.03	
Disease duration (months)	64.29 (68.73)	64.53 (32.13)	0.19	
EDSS	1.68 (1.02)	3.87 (1.23)	< 0.0001	
**White matter lesion quantification (volbrain software)**				
Total lesion count	29.67 (21.93)	39.13 (18.41)	0.03	
Number of periventricular lesions	10.38 (4.54)	9.53(4.73)	0.52	
Number of deepwhite lesions	9.04 (8.37)	12.8 (11.26)	0.09	
Number of juxtacortical lesions	8.29 (12.88)	15.07 (16.27)	0.05	
Number of infratentorial lesions	1.96(1.57)	1.73 (2.05)	0.36	
Number of cerebellar lesions	1.46 (1.50)	1.47(1.96)	0.60	
Number of medular lesions	0.50 (0.65)	0.27 (0.59)	0.24	
Total volume of lesions	0.59 (0.42)	1.12 (0.98)	0.15	
Volume of Periventricular lesions (mm^3^)	0.75 (0.35)	0.65 (0.32)	0.45	
Volume of deepwhite lesions (mm^3^)	0.02 (0.02)	0.03 (0.03)	0.80	
Volume of juxtacortical lesions (mm^3^)	0.06 (0.06)	0.08 (0.12)	0.97	
Volume of infratentorial lesions (mm^3^)	0.02 (0.03)	0.14 (0.04)	0.19	
Volume of cerebellar lesions (mm^3^)	0.01 (0.03)	0.01 (0.03)	0.31	
Volume of medullar lesions (mm^3^)	0.01 (0.03)	0.0004 (0.001)	0.20	
**Analysis of QSM maps (three experts by IKT SNAP software)**				
Number of lesions all in FLAIR	14.09 (13.21)	19 (11.53)	0.14	
Number of rim lesions (PRL)	2.39 (2.95)	4 (5.08)	0.73	
Number Hyperintense lesions	3.56 (3.43)	3 (3.4)	0.67	
Number Hypointense	0.47 (1.08)	1.07 (0.99)	0.03	
Number Isointense	7.65 (10.13)	11 (7.41)	0.04	
Percentage of Rim lesions	15.36 (12.23)	16.41 (20.46)	0.79	
Percentage of Hyperintense lesions	30.14 (17.22)	12.76 (12.35)	0.001	
Percentage of Hypointense lesions	3.82 (7.08)	6.85 (7.49)	0.10	
Percentage of Isointense	50.67 (22.74)	63.98 (25.92)	0.08	
QSM intensity Rim lesions (ppb)	19.67 (19.07)	18.30 (23.78)	0.76	
QSM intensity Hyperintense lesions (ppb)	9.85 (22.66)	3.24 (14.07)	0.53	
QSM intensity Isointense lesions (ppb)	-30.82 (11.70)	-35.59 (12.78)	0.31	
QSM intensity hypointense lesions (ppb)	-48.76 (10.68)	-52.04 (18.97)	0.85	
QSM Intensity NAWM (ppb)	-32.04 (9.97)	-30.40 (8.06)	0.73	
Size of Rim lesions (cm^2^)	0.56 (0.32)	0.42 (0.19)	0.18	
Size of Hyperintense lesions (cm^2^)	0.47 (0.37)	0.24 (0.26)	0.01	
Size of Hypointense lesions (cm^2^)	0.10 (0.05)	0.037 (0.02)	0.20	
**Normalized values [according to intracranial cavity (TIV)] of cortical/ subcortical volumes and cortical thickness**				
	RRMS	PPMS	p-value	
	Mean (± SD)	Mean (± SD)		
**MRI Analysis by Computational Anatomy Toolbox (CAT)**				
Global cortical thickness (cm)	2.34 (0.09)	2.27 (0.10)	0.03	
Grey matter total volume (mm^3^)	52.92 (1.29)	51.76 (1.02)	0.005	
**Cortical and subcortical quantification (volbrain software)**				
Cortical volume (mm^3^)	41.54 (1.36)	41.05 (1.15)	0.21	
Thalamus (mm^3^)	1.10 (0.08)	1.02 (0.12)	0.04	
Accumbens (mm^3^)	0.06 (0.01)	0.06 (0.012)	0.15	
Amygdala (mm^3^)	0.15 (0.1)	0.14 (0.02)	0.36	
Basal forebrain (mm^3^)	0.05 (0.01)	0.05 (0.01)	0.56	
Caudate (mm^3^)	0.43 (0.04)	0.40 (0.04)	0.08	
Hippocampus(mm^3^)	0.51 (0.04)	0.46 (0.04)	0.04	
Pallidum (mm^3^)	0.22 (0.09)	0.19 (0.03)	0.004	
Putamen (mm^3^)	3.27 (12.92)	0.58 (0.07)	0.03	
Ventral DC (mm^3^)	0.67 (0.04)	0.04 (0.06)	0.06	
Parahippocampal gyrus (mm^3^)	0.52 (0.04)	0.52 (0.04)	0.88	
**Analysis of leptomeningeal enhancement (LMCE)**				
Presence (1) or absence (0) of LMCE	Yes (9), no (15)	Yes (8), no (7)	0.42	
Number of LMCE	0.62 (0.96)	1 (1.2)	0.33	
**Biological analysis of serum samples from MS patients**				
CXCL13 (pg/ml)	119.3 (61.17)	89.12 (35.76)	0.35	
IL-21 (pg/ml)	331.2 (138)	284.2 (36.50)	0.47	
**Disease-modifying therapy in RRMS and PPMS group**				
	RRMS	PPMS		
Disease-modifying therapy (n)	Naive (7) Ocrelizumab (11) Ofatumumab (2) Glatiramer acetate (2) Rituximab (1) Fingolimod (1)	Naive (8) Ocrelizumab (4) Glatiramer acetate (1) Azathioprine (1) Mychophenolate mofetil (1)	N/A	

PPMS = primary progressive multiple sclerosis, RRMS = relapsing-remitting multiple sclerosis, SD = standard deviation, EDSS = Expanded Disability Status Scale, Ventral DC = Ventral Diencephalon, GM = Grey matter, FLAIR = Fluid-attenuated inversion recovery, PRL = Paramagnetic Rim Lesion, QSM = quantitative susceptibility mapping, NAWM = Normal Appearing White Matter, better CXCL13 = Chemokine (C-X-C motif) ligand 13, IL-21 = Interleukin-21, n = number of patients, N/A = not applicable, LMCE = leptomeningeal enhancement

### MRI acquisition and data analysis

Participants were examined, from September 2021 to September 2023, on a Philips Achieva TX 3 Tesla MRI Scanner (Best, the Netherlands) equipped with an eight-channel head coil. The QSM maps resulted from processing the multi-echo 3D-T1-weighted (3D-T1w) fast field echo (FFE) imaging sequence using morphology-enabled dipole inversion with an automatic uniform cerebrospinal fluid zero reference algorithm (MEDI + 0) [24].

The protocol consists of the following anatomical images: (a) 3D-T1w turbo field echo (TFE) sequences (repetition time (TR)/echo time (TE): 9.9 ms/3.7 ms, flip angle: 7°, voxel size: 0.9 × 0.9 × 1 mm, pixel banwidth (BW): 183 Hz, parallel imaging with RL acceleration factor (SENSE) 2, number of averages (NSA): 1, scanning time 5 min and 59 s, sagittal plane), (b) 3D-turbo spin-echo T2-weighted (T2w) FLAIR sequences (TR/TE: 9000 ms/600 ms, inversion time: 2420 ms, flip angle: 90°, voxel size: 1.0 × 1.0 × 1.0 mm, pixel BW: 256 Hz, NSA: 2, scanning time: 9 min and 45 s, SENSE RL and AP: 1.5 and 2 respectively, sagittal plane). (c) 3D-T1w FFE sequence (TR/TE: 38ms /45 ms, flip angle: 20°, pixel BW: 333 NSA 1, voxel size: 1.0 × 1.0 × 1.0 mm, scanning time: 9 min and 4 s, SENSE RL: 2, transverse plane).

Both 3D-T1w TFE and 3D-FLAIR sequences were acquired pre- and post-contrast administration; the post-contrast 3D-T1w (3D-T1wGd) and 3D-FLAIR (3D-FLAIRGd) were acquired 5 and 12 min, respectively, after the intravenous bolus of 0.1 mmol/kg infusion of gadoterate meglumine (Dotarem).

The susceptibility value and QSM-based size of lesions were estimated by using a region of interest (ROI) via a semiautomatic tool in IKT-SNAP segmentation software (expressed in cm^2^). We manually excluded the veins. The susceptibility of a lesion was defined as the mean of all voxels in the ROI. For lesions with rims, we evaluated the QSM intensity in the rim and not in the whole lesion.

The hyperintense FLAIR MS lesions were further evaluated in QSM maps based on their intensity range (expressed as parts per billion, ppb): (a) iso-intense (lesions with QSM maps that did not differ in intensity from the surrounding tissue) (b) hypo-intense lesions; (c) hyperintense lesions (all over the lesion); (d) paramagnetic rim-lesions PRL (lesions with hyperintense rim, along with a iso/hypointense center in QSM maps).

LMCE or foci were identified as hyper-intensities on 3D-FLAIR Gd, without corresponding hyper-intensities on 3D-FLAIR and 3D-T1w Gd sequences; we included in our analysis LMCE which were confirmed on all three planes (axial, coronal, and sagittal). Image analysis of the type of lesions and LCME was performed independently by three neurologists (A.G.V., D.K., M.B.).

Also, we analyzed the presence and subtypes of CCL (Cerebral cortical lesions). The detection of CCLs required high signal on 3D-FLAIR and low signal on 3D-T1w in pre-contrast sequences, with each lesion ranging at least 3 mm in their long axis [25]. Due to the limitations of the 3-Tesla scanner, the four neuropathological types of CCLs (1–4) according to the classification of Bø et al. were divided into two subgroups based on their topographic location: (a) group IC–SP, including IC (intracortical) and SP (subpial) lesions and (b) group LC, including leukocortical lesions [26].

### Volumetry and surface-based morphometry analysis

Volumetric data were extracted using the volBrain (http://volbrain.upv.es) platform. VolBrain software contains advanced pipelines and automatically provides volumetric information of the brain MR images at different scales [27]. The brain was segmented into whole white matter, cortex, deep gray matter structures, and ventricles. The estimation of the cortical surface was conducted using an automated processing pipeline implemented in the Computational Anatomy Toolbox (CAT, version 12.8.2) within SPM12 while running MATLAB 9.5 (R2018b; MathWorks, Natick, MA, USA). The central surface as well as cortical thickness are estimated in one step using a projection-based distance measure [28]. The vertex-wise cortical thickness measures were resampled and smoothed using a 15-mm FWHM Gaussian kernel. Intracranial cavity (TIV) volume normalization, gender and age were used as covariates. Regional differences were compared using a general linear model with the threshold value *P* < 0.01 and a family-wise error (FWE) correction.

### Biological analysis

The cerebrospinal fluid (CSF) test included white blood cell (WBC) count, total protein level, glucose level, IgG index (the normal IgG-index reference was < 0.65), and oligoclonal band (OCB) evaluation. CSF samples, after being centrifuged immediately, were stored frozen at 80^oC^ until analysis. CXCL13 and IL-21 levels were measured using commercially available ELISA kits (Thermo Fisher Scientific) according to the manufacturer’s instructions.

### Statistical analysis

Normally distributed continuous variables are presented as mean (SD) and categorical variables as frequencies (percentage). Comparisons of continuous variables were performed using the Pearson correlation test (for normal distribution) and the Spearman correlation test (for skewed distribution). A G- test was conducted to determine whether significant differences existed among all categorical variables. The G-test was selected over the Chi-Square test because it is less sensitive to small, expected frequencies. Comparisons of continuous variables between groups were performed using the independent samples t-test test (for normal distribution) and the Mann-Whitney test (for skewed distribution). Monte Carlo methods can be used to simulate the p-value, based on random sampling. This approach can be more accurate when the sample size is small, or the data are unbalanced. The effect size is given in brackets: in the case of qualitative variables, it is Cramer’s V; for quantitative variables, it is Wendt’s formula (r). Regarding Wendt’s formula, a Small Effect Size is indicated by|r| around 0.1, a Medium Effect Size by|r| around 0.3, and a Large Effect Size by|r| around 0.5 or higher. A value of 0 signifies no difference; positive values suggest that the first group tends to be larger than the second, while negative values indicate that the second group tends to be larger than the first. As for Cramer’s V: V < 0.2 indicates a weak result, 0.2 < V < 0.6 denotes a moderate result, and V > 0.6 signifies a strong result.

Multi-stage method (MM) Robust Regression was performed to investigate the associations between PRL and various MRI metrics (PPMS & RRMS). The least squares method (OLS) regression is a method that is very sensitive to outliers and for this very reason, this method was not used. On the other hand, Robust regression is a technique that can reduce the impact of outliers.

To compare the groups before and after treatment, paired tests were used. For quantitative variables: the Paired t-test was applied to variables with symmetric distributions, and the Wilcoxon Signed-Rank Test was used for those with asymmetric distributions. For the qualitative variables, McNemar’s test was conducted. All reported p-values were based on two-sided tests. Python programming language was used for all calculations.

## Results

### A. quantitative susceptibility mapping

#### White matter lesion categorization based on lesion characteristics in QSM maps

**Fig. 1:**
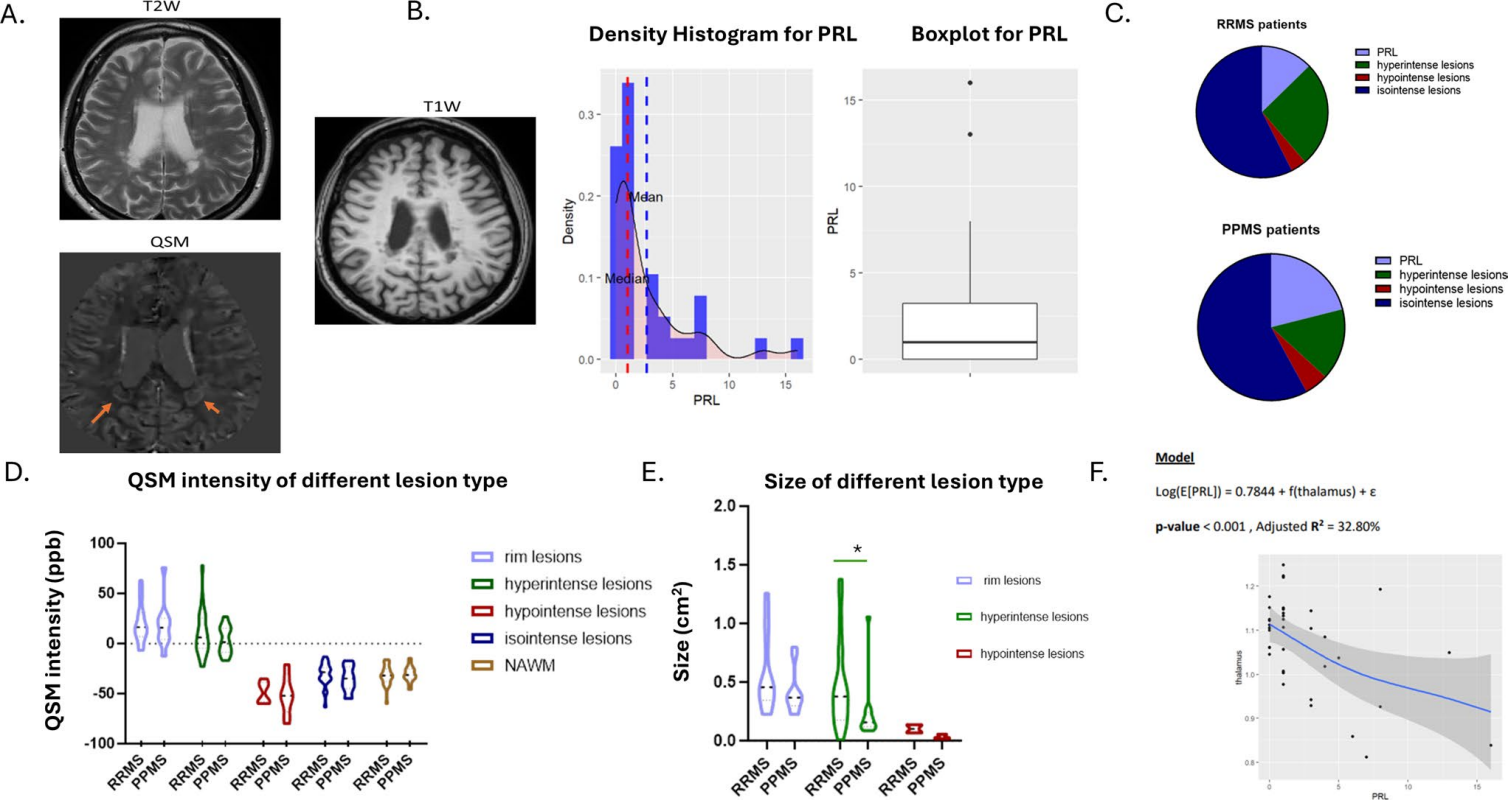
Quantitative susceptibility measures in QSM lesion types across MS disease subtypes and MRI correlations. **(A)** QSM lesion type. **(B)** Histogram of the number of PRL lesions in patients of the cohort, **(C)** Distribution of QSM lesion types (%) in RRMS and PPMS. **(D)** QSM intensity values among QSM lesion types in RRMS and PPMS. **(E)** QSM volume lesion among QSM lesion types in RRMS and PPMS. **(F)** Generalized Additive models (GAMs) of the thalamus and number of PRLs. *p < 0.05, PRL; Paramagnetic rim-lesions, QSM; quantitative susceptibility mapping, NAWM; normal-appearing white matter, PPMS; primary progressive MS, RRMS; relapsing remitting MS

The mean number of brain FLAIR hyperintense white matter lesions per patient identified was 16, and none of the patients had evidence of Gd-enhancing lesions. The WMLs (white matter lesions) (*n* = 609) showed distinct characteristics within QSM maps and were classified as iso-intense lesions (*n* = 341, 55%), hypo‐intense lesions (*n* = 26, 4.2%), hyperintense lesions (*n* = 127, 21%), and rim + lesions (*n* = 115, 18%) (Fig. 1A-C). The number of QSM rim + lesions per patient ranged from zero to a maximum of 16 (mean = 3, SD;0.9). 10 out of 38 patients (26.3%) did not have any rim + lesions (Table-1).

Further, hypo-intense lesions in QSM images showed lower absolute magnetic susceptibility than iso‐intense lesions (*p* = 0.0002), iso‐intense lesions showed lower absolute magnetic susceptibility than hyperintense lesions (*p* < 0.0001), and hyperintense lesions showed lower absolute magnetic susceptibility than PRLs in the rim region (*p* = 0.02) (Fig. 1D and Supplementary Fig. 1).

#### Comparison of lesion size across QSM lesion types

PRL lesions were larger (as measured in QSM map) and showed the highest signal intensity in QSM than all other types of lesions. Hypo-intense lesions were smaller than hyperintense lesions (*p* = 0.0008), which in turn were smaller than PRLs (*p* = 0.007) (Fig. 1E and Supplementary Fig. 1).

#### Comparison of QSM lesion type frequency between RRMS and PPMS

There was no difference in the frequency of rim-lesions between patients with RRMS and those with PPMS (Table 1; Fig. 1C). In RRMS, an average of 2.4 rim + lesions (or PRL) were detected, whereas 4 rim + lesions were detected in PPMS (Table 1). The hyperintense lesion size (expressed as cm^2^) as well as the percentage of hyperintense lesions in RRMS were greater than in PPMS (*p* = 0.01 and 0.001, respectively) (Fig. 1E; Table 1). Moreover, a trend was observed toward fewer isointense (absolute number and percentage) lesions in RRMS than in PPMS (*p* = 0.04, *p* = 0.08, respectively) (Table-1). There was no difference in the QSM intensity of NAWM between RRMS and PPMS patients (Fig. 1D).

### B. Leptomeningeal contrast enhancement and cortical lesions

**Fig. 2:**
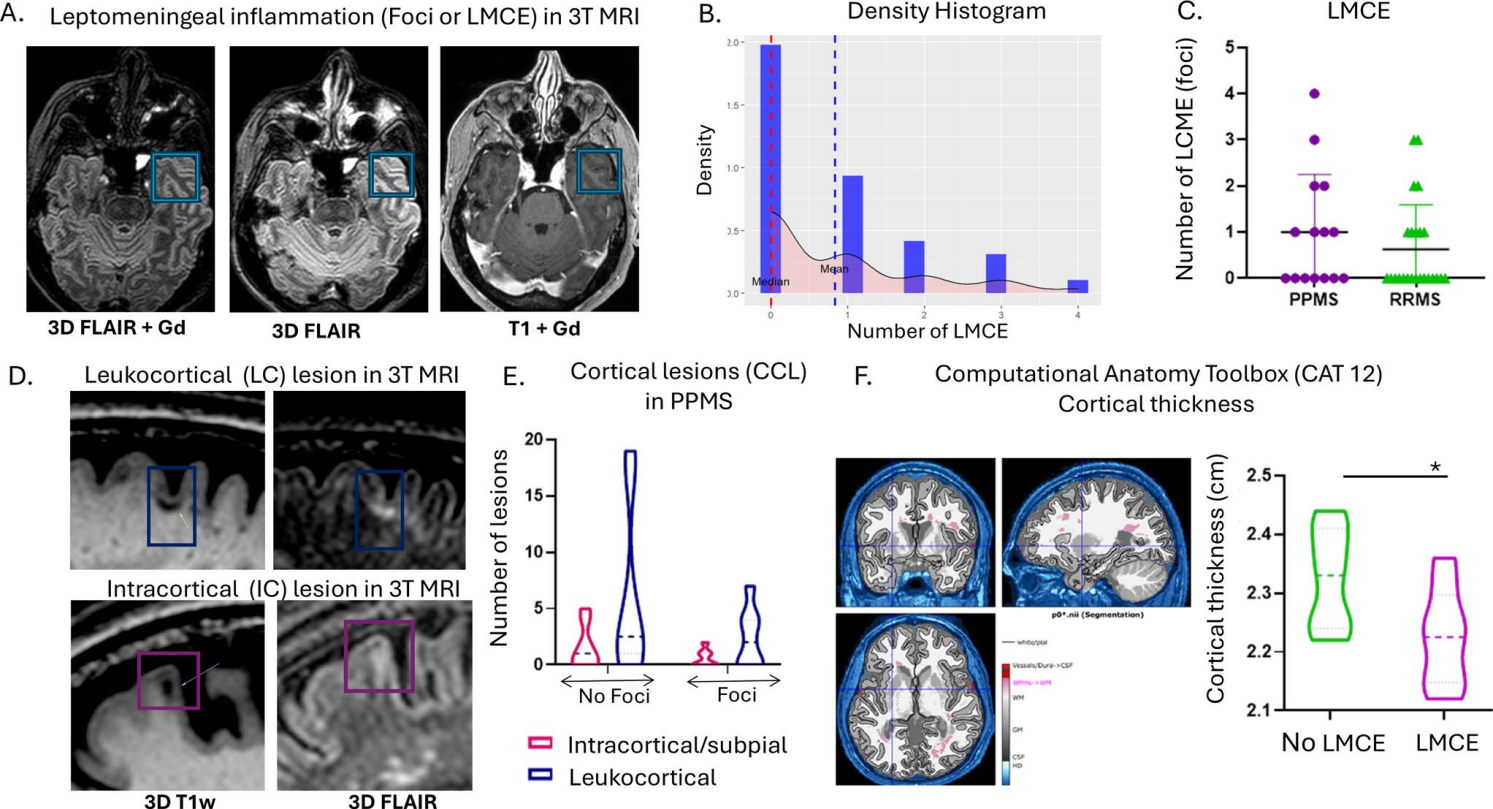
Leptomeningeal enhancement and cortical thickness association in MS patients. **(A)** Leptomeningeal contrast-enhancement in an MS patient. From left to right, LMCE are visible on 3T postcontrast T2-FLAIR images, but not on pre-contrast T2-FLAIR (middle) or postcontrast T1-weighted images. **(B)** Histogram of the number of LMCE of patients in the cohort. **(C)** Number of LMCE in RRMS versus PPMS. **(D)** Presence of leukocortical and intracortical type of cortical lesion in 3T MRI 3D T1w and 3D FLAIR. **(E)** Cortical lesions (both leukocortical and intracortical/subpial) in patients with PPMS stratified based on the presence of LMCE. **(F)** Representative image from an MS patient derived for SPM12 CAT 12.8.2. Cortical thickness is presented in patients with and without foci. PRL; Paramagnetic rim-lesions; PPMS, primary progressive MS; RRMS, relapsing remitting MS; ppb, parts per billion, LMCE; leptomeningeal contrast-enhancement, LC; leukocortical lesions, IC; intracortical lesions, CCL; Cerebral cortical lesions, CAT; Computational Anatomy Toolbox

LMCE was assessed in 3D-FLAIR post-gadolinium (3D-FLAIRGd) sequences (Fig. 2A-B). The presence of LMCE was detected in 37.5% of patients with RRMS and 53% of PPMS patients (Fig. 2C).

In the RRMS disease subgroup (*n* = 21), the number of LMCE had a statistically significant negative relationship with the presence of PRL [0.018 (*r* =- 0.51)]. Negative Binomial Regression analysis further strengthened that association as it showed that the estimation of PRL on LMCE = 0.53, CI: [0.25, 0.90], p-value = 0.048, meaning that the estimated effect of PRL on the number of LMCE is significant, with an incidence rate ratio (IRR) of 0.53 (Supplementary Material–General Additive Models). In the whole MS cohort, no correlation was found among LMCE and PRL.

We further evaluated in PPMS the type of cortical lesions (leukocortical and intracortical/subpial). The detection of intracortical/subpial (IC) and white-cortical (leukocortical) lesions (LC) was performed using the 3D-T1w sequence and FLAIR sequences (Fig. 2D). We found that patients with the presence of at least one LMCE showed only a trend, toward more total cortical lesions. The mean number of IC and LC in patients with at least one LMCE was 1.6 and 6.1, respectively, whereas in patients without LMCE was 0.57 and 2.4, respectively (Fig. 2D-E and Supplementary Fig. 2A).

### C. MRI volumetry and association with lesion types

#### Association between QSM rim-lesions (PRL) and Gray matter damage

The mean global cortical thickness was lower in PPMS than in RRMS (*p* = 0.03). Regarding differences in deep gray matter fractions, PPMS showed slight thalamic (*p* = 0.04), pallidum (*p* = 0.004) and putamen (*p* = 0.03) atrophy and displayed higher disability scores than RRMS (*p* < 0.001) (Table-1).

We aimed to investigate the relationship of chronic active rim + lesions, identified as a high-intensity ring on QSM, with the clinical severity, disease type (RRMS and PPMS), and imaging markers of neurodegeneration.

In the entire mixed cohort of MS patients (*n* = 36), by applying robust linear regression, we found a positive correlation between PRL and disease duration in months (coef;0.017, *p* = 0.013) and various MRI features indicative of high lesion burden (e.g. volume of periventricular lesions; coef;2.97, *p* < 0.01 and Supplementary Tables 1–2). Moreover, we found a negative correlation of PRL with various MRI parameters indicative of neurodegeneration (e.g. volumes of thalamus; coef;-14.88, *p* < 0.01, Fig. 1F and Supplementary Tables 1–2). We further generated General Additive Models (GAMs), showing that volumetric analysis of the thalamus and pallidum exhibited the most significant effects on PRL (Supplementary Material–General Additive Models).

In the PPMS group (*n* = 15), the number of PRLs was significantly correlated with the volume of periventricular lesions (coef;3.09, p-value < 0.001), volume of the thalamus (coef;-19.01, p-value < 0.001), and volume of the pallidum (coef;-114.5, p-value < 0.001). There was a strong relationship between RRL and the number of juxtacortical lesions (coef;0.13, *p* = 0.006) (Supplementary Tables 3–4). In the RRMS group, the PRLs were correlated with the number of juxtacortical lesions (coef;0,13, p-value < 0.0001) and disease duration (coef;0.016, p-value < 0.001) (Supplementary Tables 5–6).

**Fig. 3:**
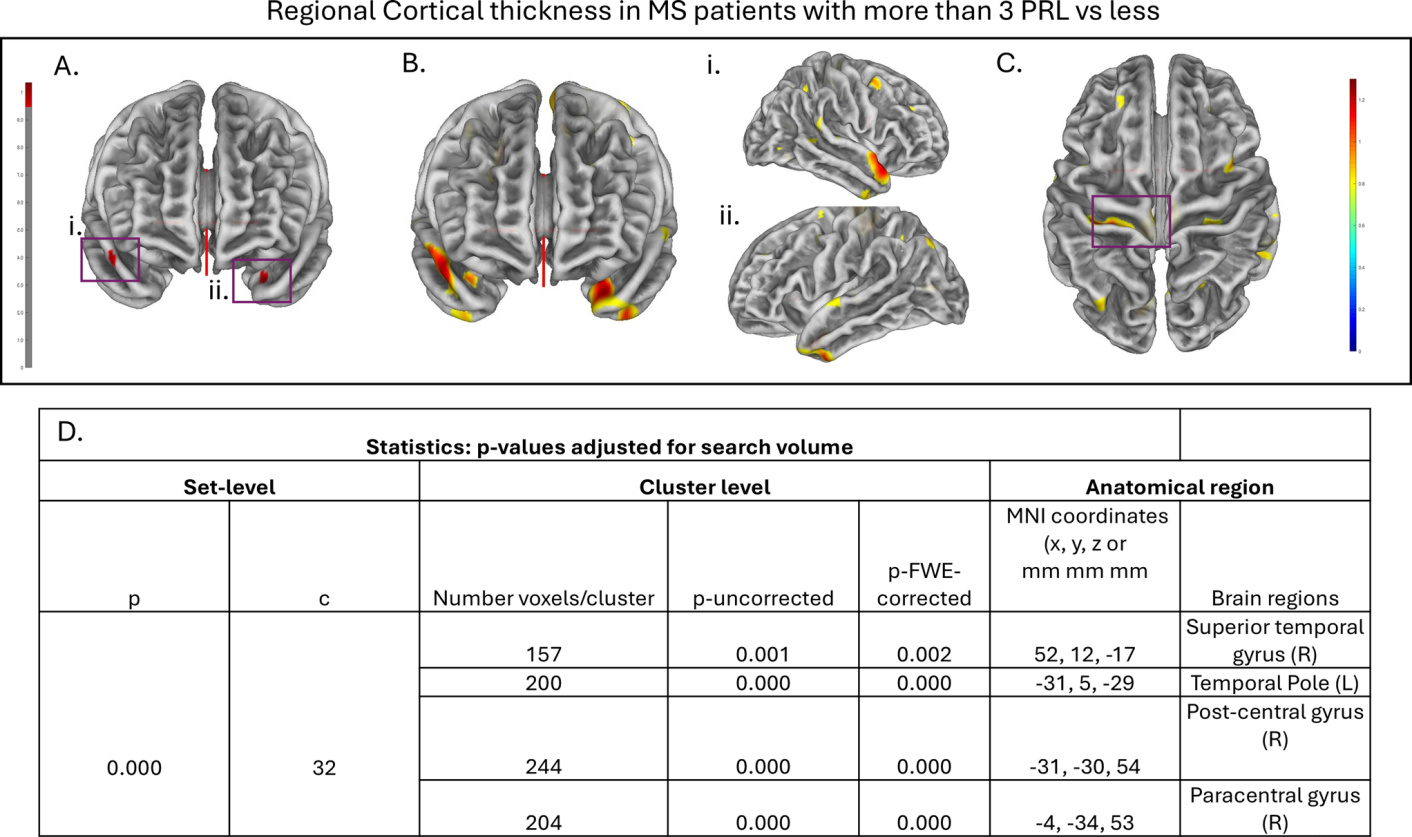
Regional cortical thickness assessment by surface-based morphometry (SBM) analysis. **A-C**. Areas of significant cortical thickness differences in MS patients with > 3 PRL lesions vs. 3 PRL, threshold at *p* < 0.001 with family-wise error (FWE) correction for multiple comparisons at the vertex level, and with minimum extent cluster size correction (K = 21) at the cluster level. Cranial and lateral views are depicted. **A.** two areas with statistically significant difference in cortical thickness located in the (i) superior temporal gyrus and (ii) the temporal pole (p-FWE-corrected). **B.** The same regions are depicted as larger cluster areas (p-uncorrected). **C.** Another two regions with statistically significant difference are located in the post- and paracentral right gyrus (Right). **D.** Locations of significance, according to their MNI coordinates, based on Automated Anatomical Labeling and Yale BioImage Suite brain atlases. MNI, Montreal Neurological Institute. FEW, family-wise error; PRL, paramagnetic rim; R, right; L, left

In the entire MS cohort, patients with more than 3 PRL had less regional cortical thickness in 3 areas (right superior temporal gyrus, left temporal lobe and post/paracentral area left) (age and the total intracranial cavity were used as covariates for normalization) (Fig. 3). 

Collectively, our findings demonstrate that chronic-active lesions, as identified in the QSM sequence by the presence of a hyperintense rim and hypo/isointense core, are associated with the extent of white matter lesions (especially juxtacortical lesion burden), regional cortical thickness, and subcortical gray matter atrophy, particularly in PPMS.

The other types of QSM lesions were less associated with gray matter volumes or cortical thickness. The only significant correlations were found among the total number of hyperintense lesions and the volume of thalamus (*r*=-0.432, *p* = 0.007), more evident in PPMS (*r*=-0.649, *p* = 0.014) and among the number of isointense lesions and the total cortical thickness (*r*=-0.451, *p* = 0.0045) (Supplementary Fig. 3).

#### Association between leptomeningeal LMCE and Gray matter damage

We further investigated the relationship between leptomeningeal enhancement and clinical severity, disease type (RRMS and PPMS), and imaging markers of neurodegeneration.

In the PPMS group (*n* = 15), the number of LMCE had a statistically significant relationship with “cortical thickness” [0.038 (*r* =-0.54)] and “age at disease diagnosis” [0.046 (*r* = 0.52)] and marginally with EDSS (Expanded Disability Status Scale) [*p* = 0.088 (*r* = 0.88)]. Patients with more than one LMCE had a lower total cortical thickness (Fig. 2F, Supplementary Tables 7 and Supplementary Material–General Additive Models).

Moreover, there was no strong correlation between cortical thickness and total number of cortical lesions (CCL = LC + IC) (*r*=-0.42, CI: -0.77, *p* = 0.12) (Supplementary Fig. 2B and data not shown).

#### EDSS correlates and correlation matrix for various clinical and MRI parameters

In the whole group of MS patients, the EDSS showed correlations with gray matter total volume, the volume of pallidum, the percentage of hyperintense lesions and the volume of hyperintense lesions (all *p* < 0,05). In the RRMS groups, EDSS correlated with the percentage of isointense lesions (*p* = 0.023) and the volume of deep white matter lesions (*p* = 0.039). In the PPMS group, no significant correlations were found (Supplementary Table 8).

Serum levels of the cytokines CXCL13 and IL-21 did not exhibit any significant correlation with disease parameters or MRI biomarkers in the entire study cohort. PRL lesions in PPMS were associated with cytokine CXCL13 levels (*r* = 0.73, *p* = 0.05). Other important associations were observed between QSM intensity in NAWM and the volume of the thalamus and pallidum (for both *r*=-0.47) (Supplementary Fig. 4).

### D. Predictors of cortical thickness in Ms patients

**Fig. 4:**
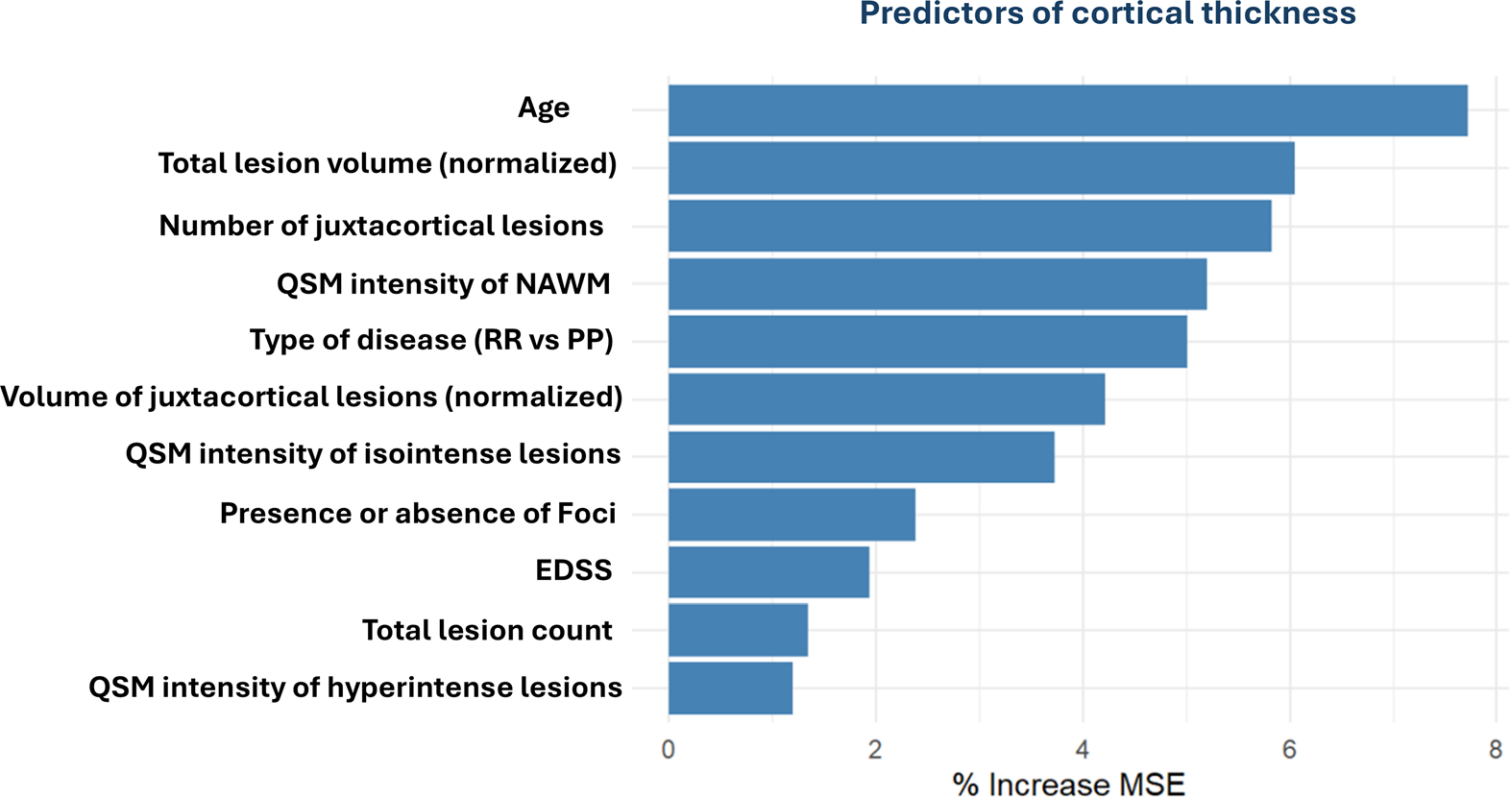
Random Forest informative predictors of cortical thickness in MS patients. Distribution of variable importance of demographic, clinical, and MRI features to explain cortical thickness. EDSS = Expanded Disability Status Scale; MS = multiple sclerosis; QSM = quantitative susceptibility mapping; NAWM, normal appearing white matter

Random Forest is an ensemble machine learning method known for its robustness and accuracy, that involves building multiple decision trees and combining their outputs. The diagram in Fig. 4 presents the variable importance as determined by the random Forest model for predicting cortical thickness. This indicates that the increasing age, total lesion volume (especially juxtacortical lesions), QSM parameters (like: QSM intensity of NAWM), type of disease (PPMS vs. RRMS) and the presence of LMCE are the most significant predictors. The Percent Increase in Mean Squared Error (%IncMSE) presents the importance of a feature: the larger the increase, the more important the feature is to the model’s predictive accuracy.

### E. Longitudinal evaluation of lesions and assessment of the role of B cell depleting therapies

**Fig. 5:**
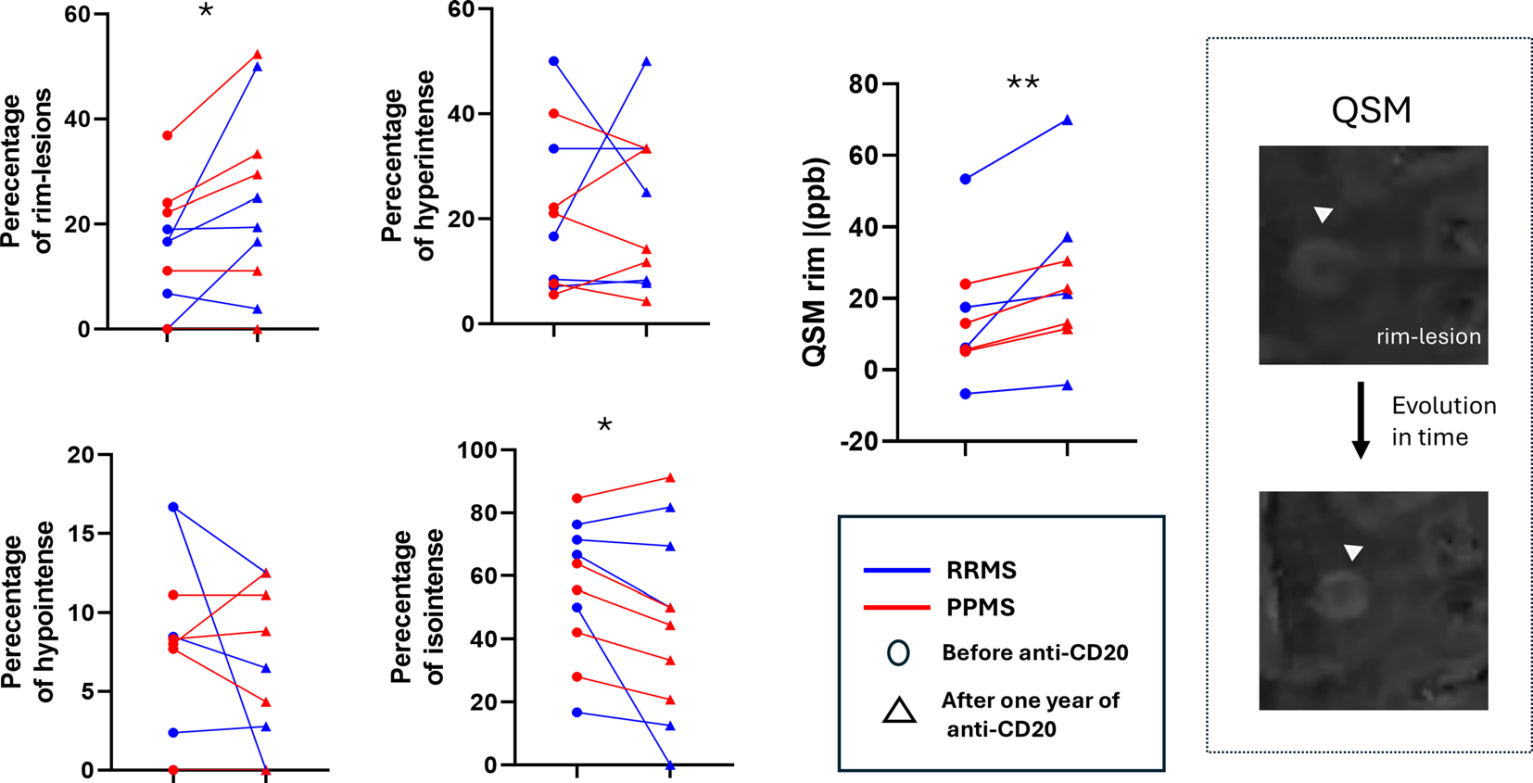
Effect of anti-CD20 treatment in QSM lesion types. Paired analysis of QSM lesion type before and after anti-CD20 therapy. Representative MRI features of a rim-lesion in a RRMS patient during the one year follow up. QSM = quantitative susceptibility mapping, ppb = parts per billion

Ocrelizumab was administered for a mean time of 13.98 months (SD:12.22) in eleven patients (5 PPMS and 6 RRMS), and they underwent two MRI scans during follow-up to assess specific radiological features. Comparing values before and after treatment, we found that patients did not manifest significant clinical deterioration (mean EDSS before anti-CD20; 2.4, after anti-CD20; 2.7) and the total number of lesions remained stable after 1.112 years of follow-up (SD: 0.94). Also, patients after treatment showed an increased percentage of PRL lesions (p-value = 0.015). Interestingly, the QSM intensity in the rim of lesions increased (p-value = 0.0078) (Table 2; Fig. 5). No difference was found regarding the number of LMCE (Table 2).

**Table 2:** Clinical and MRI characteristics before and after Anti-CD20 treatment

	Before treatment	±SD	After treatment	±SD	*p*-value
Number of periventricular lesions	11	4.12	9.55	3.36	0.04
Gray matter total volume (mm^3^)	754.01	65.06	751	63	0.06
Cortical volume(mm^3^)	597.54	54.65	594.24	53.59	0.06
GM (mm^3^)	646.5	67.34	639.45	61.14	0.07
Cortical thickness	2.28	0.11	2.27	0.09	0.14
Number of PRL	3.63	3.34	4.91	3.91	0.05
Number of hyperintense lesions	3.38	2.97	3.91	2.34	0.10
Number of hypointense lesions	1.88	1.46	1.36	1.63	0.10
**Percentage of PRL**	14.56	8.12	24.12	16.93	**0.01**
Percentage of isointense lesions	55.52	21.61	45.37	29.72	0.04
**QSM intensity of rim (ppb)**	14.76	18.11	25.28	22.05	**0.008**

SD = standard deviation, EDSS = Expanded Disability Status Scale, Ventral DC = Ventral Diencephalon, GM = Gray matter, PRL = Paramagnetic Rim Lesion, QSM = quantitative susceptibility mapping, ppb = parts per billion

**Fig. 6:**
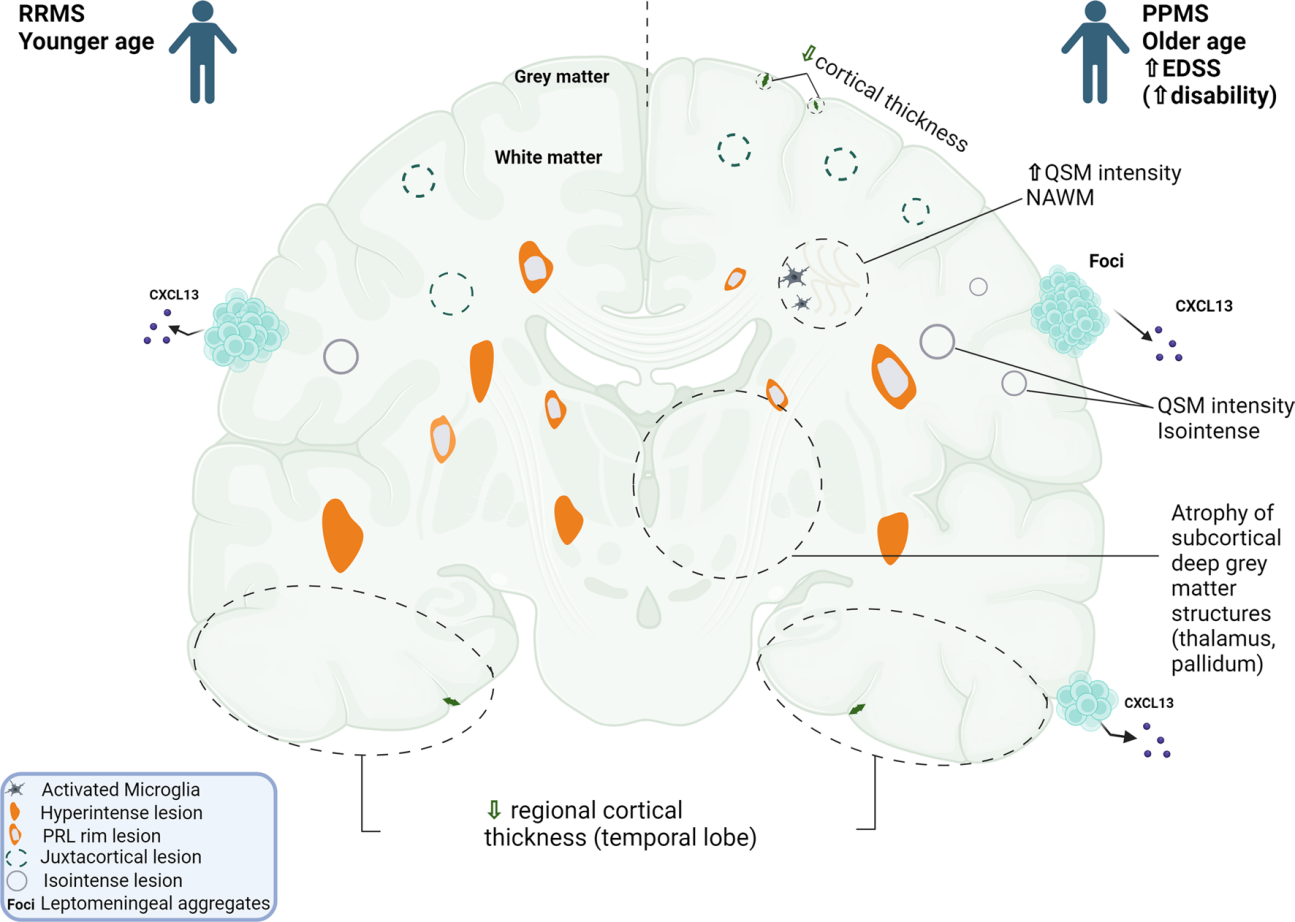
Cortical thinning in Multiple Sclerosis is of multifactorial origin. Apart from age and disease duration that are main drivers of neurodegeneration, other MS related parameters contribute to cortical damage and possibly to neuronal injury. Novel MRI markers applied in this study captured evidence of cortical tissue damage both in RRMS and PPMS. In PPMS, cortical thickness was found to associate with leptomeningeal inflammation (LCME or foci) and specific pathology originating from the white matter (either normal-appearing or not) especially the higher presence of juxtacortical lesions, and the QSM intensity of isointense lesions and NAWM. The presence of at least 3 rim-lesions is associated, not to overall cortical thinning, but to regional cortical thickness in specific areas like temporal lobe and was found in both RRMS and PPMS patients. NAWM; normal appearing white matter, RRMS; relapsing remitting MS, PPMS; primary progressive MS, QSM; quantitative susceptibility mapping, LCME; leptomeningeal contrast enhancement, PRL; paramagnetic rim-lesions. This image was generated by BioRender.

Figure 6 summarizes in an illustrative manner all the findings of the Random Forest and provides a summary of the results.

## Discussion

The pathobiology of compartmentalized inflammation in Multiple Sclerosis is elusive. Persistent leptomeningeal inflammation (in the form of ectopic follicles or not) and expanding chronic active lesions are among the core features of the disease driven neurodegeneration. Current treatment modalities effectively prevent waves of inflammation from the periphery to enter CNS, but are not sufficiently effective in controlling the progressive nature of the disease. Recent concepts in MS pathophysiology, have suggested the use of a surface-in model, possibly involving soluble toxic factors arising from the meninges and/or CSF and affecting the underlying white matter (subpial cortical and periventricular lesions) [29].There is limited evidence if meningeal inflammation is associated with chronic active lesions, particularly those located in periventricular regions. Herein, we found no connection among PRLs (MRI surrogate marker of chronic active lesions) and LMCE (surrogate marker of leptomeningeal inflammation and/or deficiencies in the meningeal blood-brain barrier) on 3T MRI in MS patients. In RRMS PRL correlated with disease duration and lesion burden, whereas in PPMS associated mostly with subcortical atrophy measures. We were able to identify as predictors of cortical thinning the type of disease (PPMS), the presence of LMCE, the burden of juxtacortical lesions and the NAWM QSM intensity, but not PRL. Instead, MS patients with more than 3 PRLs exhibit reduced regional cortical thickness. Our findings strongly indicate that with advanced MRI techniques in daily clinical practice we can capture different aspects of neurodegeneration in patients with both PPMS and RRMS.

PRL lesions are present in both RRMS and PPMS, suggesting that they represent as disease-specific characteristics (state biomarker). We found that 78% of RRMS and 66% of PPMS patients harbor at least one PRL. In RRMS, an average of 2.4 rim + lesions were detected per patient, whereas in PPMS, 4 rim + lesions were detected. In line with our results, a meta-analysis showed that 40.6% of MS patients have at least one PRL [30]. The presence of chronic active lesions seems to be a type of MS lesion not only found in PPMS but also RRMS patients. No correlation was found among the presence of PRL and EDSS. Especially in RRMS the number of PRL lesions correlated with disease duration. We found a negative correlation of PRL with various MRI parameters indicative of neurodegeneration (mainly volumes of pallidum and thalamus), especially in PPMS, whereas in RRMS PRL lesions were associated with lesion burden (especially juxtacortical lesions). In previous studies, patients with at least one PRL presented lower cortical gray matter volumes and higher brain atrophy rates [31, 32]. It is currently unknown what causes neurodegeneration in subcortical gray matter, especially in cases where no obvious demyelination occurs in deep gray matter. To what extent the thalamus and its neurons are vulnerable to remote lesions disrupting the connecting white matter tracts is a matter of future investigation. Some studies, among which ours, based on statistical correlations suggest an influence of total lesion burden and specifically rim-lesions on thalamic volume [33, 34].

Our work indicates the relationship between regional cortical thickness and rim-lesions. This is consistent with a recent study showing that the presence of at least one rim + lesion was related to more cortical thinning in younger patients (< 45 years) [35]. We found that patients with more than 3 PRL lesions exhibited less cortical thickness in three areas with significant differences when compared with those with less than 3 PRL. We did not notice any spatial association between temporal white matter lesion location and regional atrophy (data not shown). Our findings support the concept of spatially oriented neurodegenerative processes in MS that are more prominent in those with higher incidence of chronic active lesions [36]. Temporal cortical thinning (right superior temporal gyrus, left temporal lobe, left post/para central gyrus) could be an independent measure of ongoing severe progressive disease and not directly related to the PRLs, but caused by the same underlying biological mechanisms. On the other hand, an underlying (tract) connectivity of certain areas projecting to this part of the temporal cortex could account for the cortex pathology. Excess iron (located in the rim lesion edge) exacerbates oxidative stress, which, in turn, increases the release of inflammatory mediators by activating microglia and macrophages, resulting in demyelination and axonal damage in more remote areas. Reduction of cortical gray matter volume and thinning may be the consequence of white matter pathology via retrograde degeneration, thus explaining the significant neuronal loss observed even in the non-demyelinated cortex [37]. Previous studies have identified significantly more temporal thinning in cognitively impaired MS patients (domains related to specific cognitive functions; decision making, attention, memory and executive functioning) [38, 39]. Future studies are needed to highlight the clinical relevance of this association found in our study with the incorporation of complex cognitive tasks along with tractography (DTI) results.

Of note, rim-lesions correlated with regional thickness and not the total cortical thickness. The cortical thickness correlated only modestly with cortical volume, so we believe that regional thickness may reflect pathological mechanisms that precede the onset of cortical atrophy. A larger cohort of patients with higher disability scores or secondary-progressive MS could provide further insights. So, we postulate that regional thickness is a severity measure in early MS patients (in our cohort the mean disease duration was 5 years).

It has been shown that leptomeningeal contrast-enhancement, as determined by brain MRI, is associated with subcortical demyelination and possibly leptomeningeal inflammation in post-mortem analyses from MS patients [12, 40, 41]. In our study, there was no significant association between the presence of LMCE and cortical lesions. This finding is consistent with those of Ignani et al. who found no correlation between cortical lesions and leptomeningeal enhancement on 7-Tesla MRI in MS [19]. By applying various statistical analyses, we showed that as cortical thickness decreases, there is an associated increase in the number of LMCE only in PPMS patients. Our data collectively suggest a direct link between leptomeningeal inflammation and global thinning of the cortex in progressive MS patients.

Global cortical thickness was predominantly reduced in PPMS patients and did not show any association with cortical lesions. So, we tried to identify novel factors affecting the global cortical thickness, that is a measure of more severe neurodegeneration than regional cortical thinning. We applied Random Forest analysis to identify potential predictors of cortical thickness and found that apart from classical factors such as increasing age and type of disease (PPMS), we also found various contributing novel MRI parameters such as the QSM intensity in NAWM and QSM intensity of isointense lesions. We found that specific features [WM lesion volume (especially juxta cortical lesions), patient age, presence of LMCE, and QSM intensity in NAWM and isointense lesions] carried most of the prediction power for cortical thinning. Previous studies assessing factors predicting cortical damage have never combined all these parameters for prediction models and we believe that our results support the complexity of MS pathology across the whole brain [42, 43].

In our study, no significant associations were found between LMCE and PRLs. This suggests that the underlying pathological processes leading to these imaging findings are unrelated. We found an interesting finding that in RRMS but not PPMS, as PRL increases, the number of LMCE decreases (higher PRL is associated with a lower number of LMCE). One explanation would be the type of inflammation in the rim and LMCE are different between RRMS and PPMS (different interplay among innate and adaptive immunity factors). Previous research has demonstrated that patients with PPMS who presented leptomeningeal enhancement are characterized by an increase in the terminally differentiated mature NK (natural killer) subpopulation and a highly cytotoxic NK population in blood [44]. We did not find any significant differences in cytokine levels associated with LMCE. Meningeal ectopic lymphoid structures are reported in MS and are considered to be niches of self-organizing CXCL13-expressing inflammatory aggregates that possibly require the action of IL-21 and T follicular helper cells to support B-cell survival [45–47]. Chronic active lesions are mainly peri ventricularly located and to a lesser extent juxta cortically but could represent a niche for production of B cell homing cytokines [48]. A post-mortem study in progressive-MS, assessed the relationship between meningeal inflammation and the state of subcortical WM lesional activity and found that patients with a higher extent of meningeal inflammation harbored a greater proportion of both active and mixed active/inactive chronic WM lesions [12]. Nevertheless, no explanation for a direct pathogenetic link has been made. We cannot rule out a potential link between chronic active lesions and leptomeningeal aggregates in progressive MS patients and larger cohort studies are needed to address this.

We found no difference in the presence of LMCE in patients treated with anti-CD20 therapies. Accordingly, Dahal et al. in a small pilot trial, reported that ocrelizumab did not significantly reduce the number, the volume of leptomeningeal foci or the dural enhancement in MS patients [49]. Moreover, B cell depletion therapy does not resolve chronic active lesions another evidence of compartmentalized inflammation [50]. Interestingly, we found that compared with all other lesions, PRL showed a slight increase when expressed as a percentage compared with the total lesions, and the QSM intensity in the rim showed a substantial increase.

The longitudinal peak of QSM intensity in the rim (within one year) at follow-up can be regarded as the end effect of pathologic alterations in myelin and iron content that occur during different stages of lesion development. This finding could be attributed to persistent PRL evolution in the brain and to local changes in microglial cells/macrophages in the rim. Our findings are consistent with previous studies in which a gradual increase in lesion susceptibility appears as an early lesion evolves to the chronic state [51]. It has been suggested that the QSM increase, which occurs the first months to years of lesion development, is more likely related to degradation of the myelin debris within macrophages/microglial cells and the release of iron [52, 53]. QSM rim intensity could represent a novel future MRI biomarker for testing the development of targeted neuroprotective and reparative drugs.

One disadvantage of our study is the relatively small number of participants and the small follow up under anti-CD20 treatment. Nevertheless, we studied a high number of lesions (leptomeningeal and white matter) and characterized them with advanced MRI sequences. We also lack pathology evidence of the type of lesion in QSM (hyperintense/isointense/hypointense/rim). Pathology data are missing to measure directly the iron content in the rim during lesion evolution. Further studies are needed to unravel to which extent anti-CD20 treatment agents affect molecular mechanisms driving iron release and the expansion of chronic active lesions in MS.

## Conclusion

Our findings indicate the importance of detecting chronic active lesions at both relapsing and progressive forms of the disease and show a strong correlation between the existence of these lesions and the clinical and imaging characteristics of neurodegeneration. Our study indicates that QSM parameters such as QSM intensity of NAWM and isointense lesions as well as the presence of at least one LMCE are informative predictors of total cortical thickness. The complex interplay between the innate and adaptive immune systems contributes to different types of inflammatory responses in RRMS and PPMS patients, and this complex relationship is further reflected by novel MRI techniques that could capture novel disease aspects amenable to therapeutic interventions.

## Electronic supplementary material

Below is the link to the electronic supplementary material.

Supplementary Material 1

## Data Availability

The data that support the findings of this study are available upon reasonable request.

## Abbreviations

MS: Multiple Sclerosis
RR: Relapsing remitting
PP: Primary progressive
QSM: Quantitative susceptibility mapping
LMCE: Leptomeningeal contrast enhancement
NAWM: Normal appearing white matter
PRL: Paramagnetic iron rim
WML: White matter lesions
SP: Subpial
IC: Intracortical
LC: Leukocortical
CCL: Cerebral cortical lesions
GAMs: General Additive Models
MRI: Magnetic resonance imaging
ROI: Region of interest
Gd: Gadolinium
DTI: Diffusion Tensor Imaging
TIV: Intracranial cavity
CXCL13: Chemokine (C-X-C motif) ligand 13
IL-21: Interleukin-21
GM: Grey
CAT: Computational anatomy toolbox
OCB: Oligoclonal bands
