# Supplementary material for: Neurodegeneration correlates of iron-related lesions and leptomeningeal inflammation in multiple sclerosis clinical subtypes
Source: Neuroradiology. 2025 Mar 25;67(6):1541–55. doi: 10.1007/s00234-025-03595-0 (PMC12357800; doi:10.1007/s00234-025-03595-0)
Supplement: Supplementary file 1 — Supplementary Material 1 [file 234_2025_3595_MOESM1_ESM.docx]

**Supplementary Figures**

**
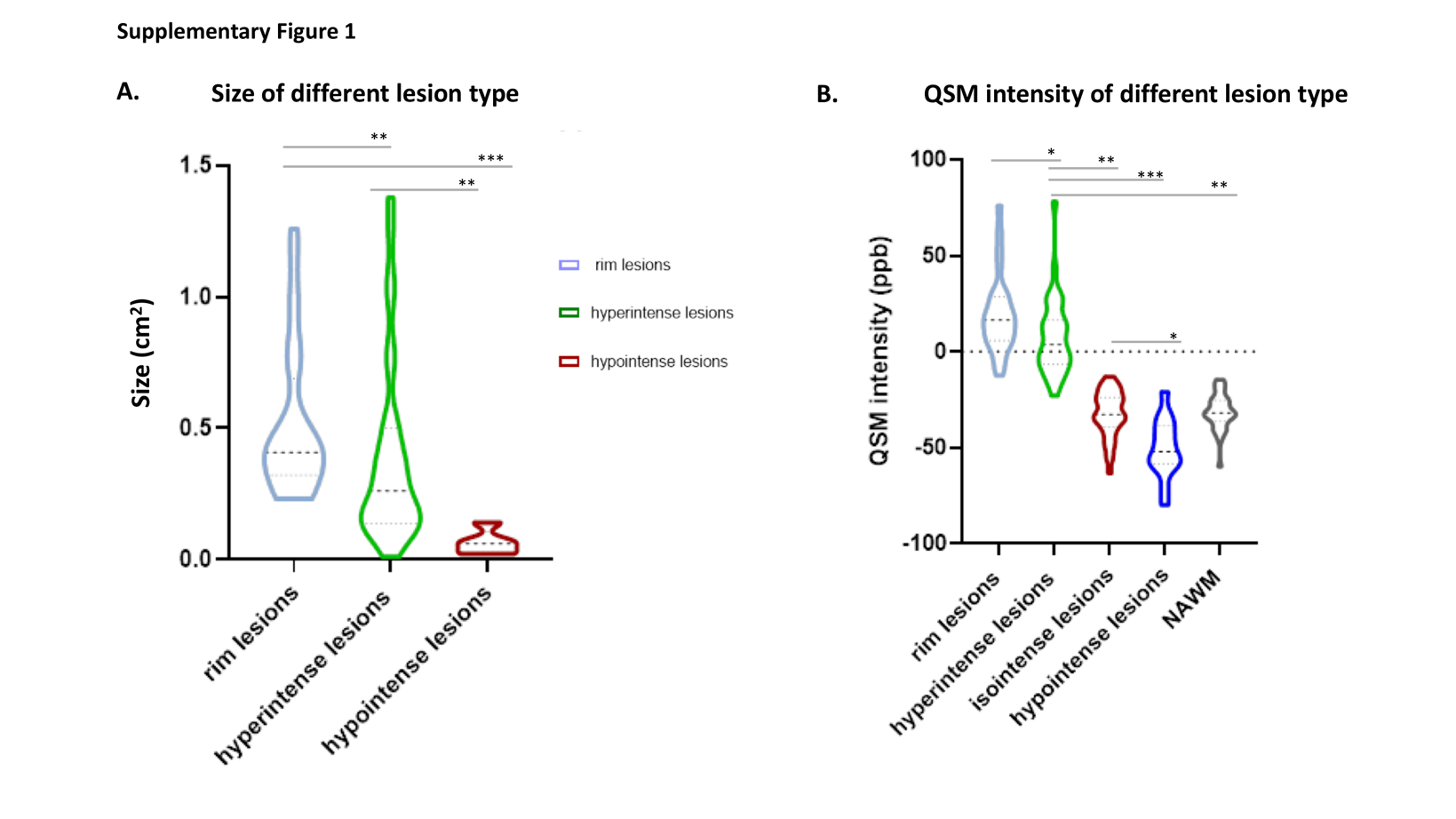
**

**Susceptibility lesion intensity and volume for different types of lesions as defined on QSM maps.** (A) Mean volume size across QSM lesion types. (B) Average susceptibility in different type of lesions (rim, hyperintense, isointense, and hypointense) and in NAWM.

* p < 0.05; **p < 0.001; ***p < 0.0001. NAWM = normal‐appearing white matter, QSM = quantitative susceptibility mapping.


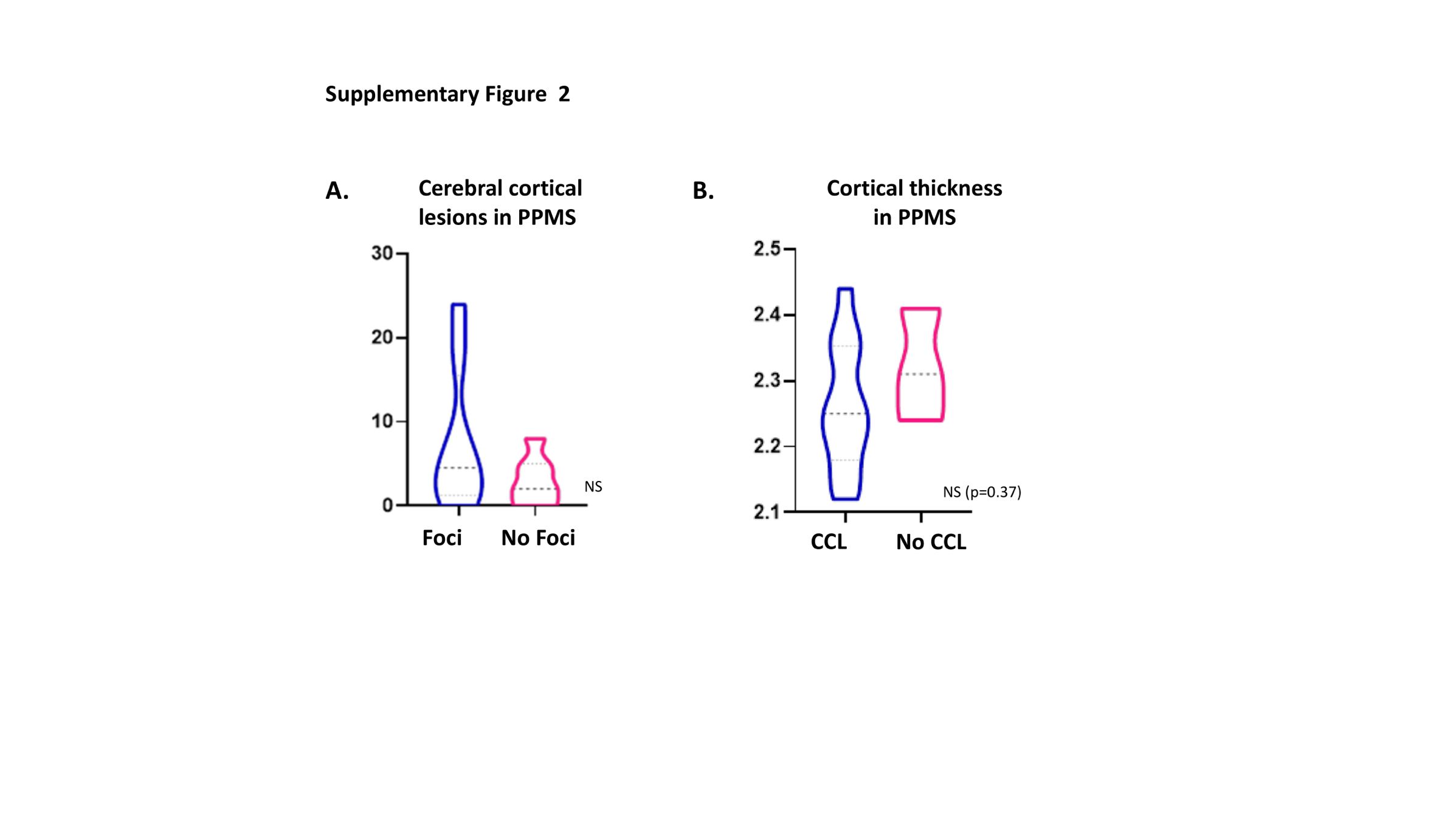


**Cerebral cortical lesions in PPMS patients.** 15 PPMS patients were stratified in two groups **(A)** based on the presence of foci, **(B)** and the presence of total cerebral cortical lesions (CCL). PPMS= primary progressive Multiple Sclerosis.


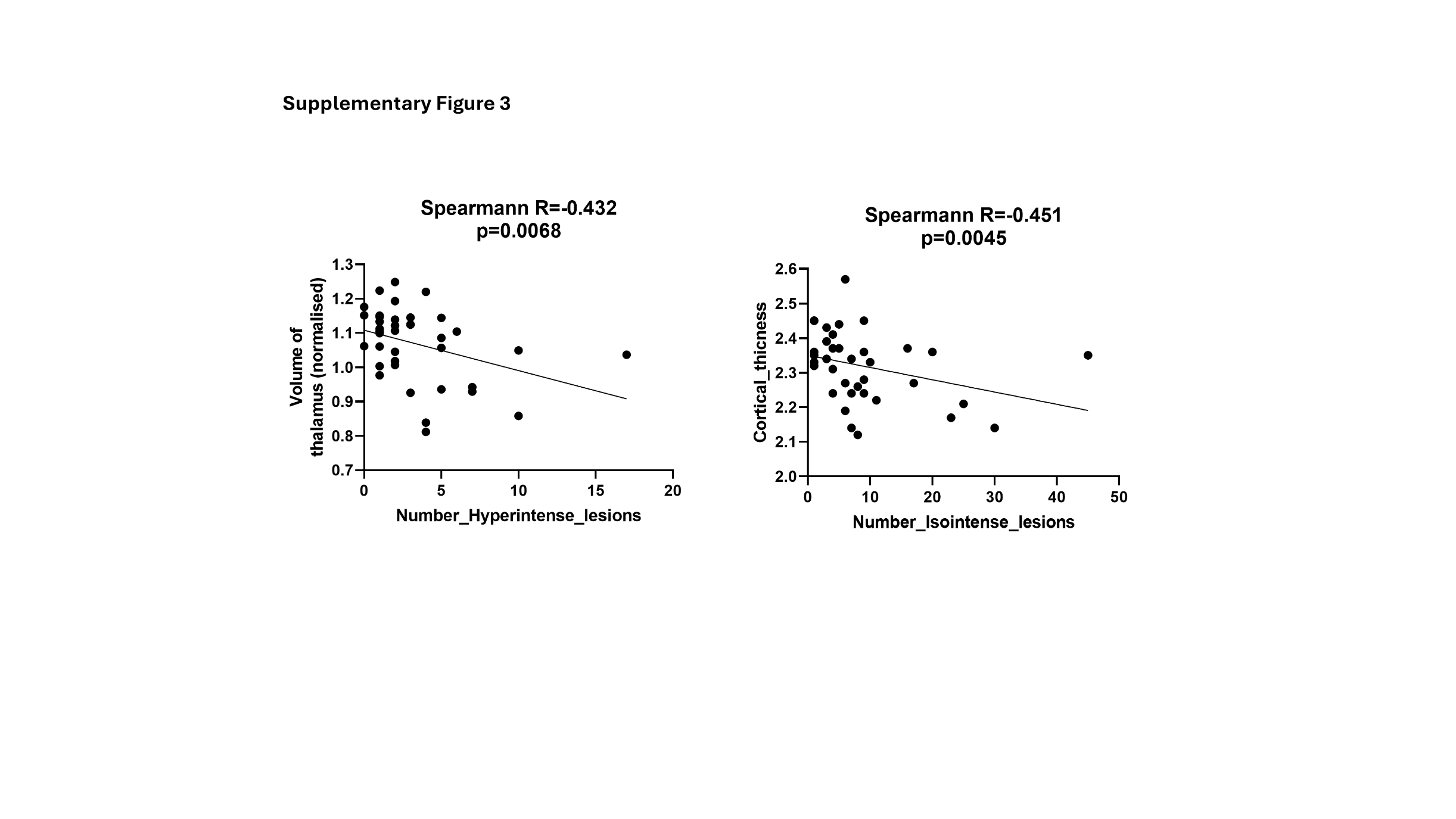


**QSM lesion types and correlation with MRI indices of neurodegeneration.** Graphs depict the correlation analysis in the total cohort of MS patients of the total number of hyperintense and isointense lesions (defined by QSM maps) with the normalized volume of thalamus and the total cortical thickness, respectively.


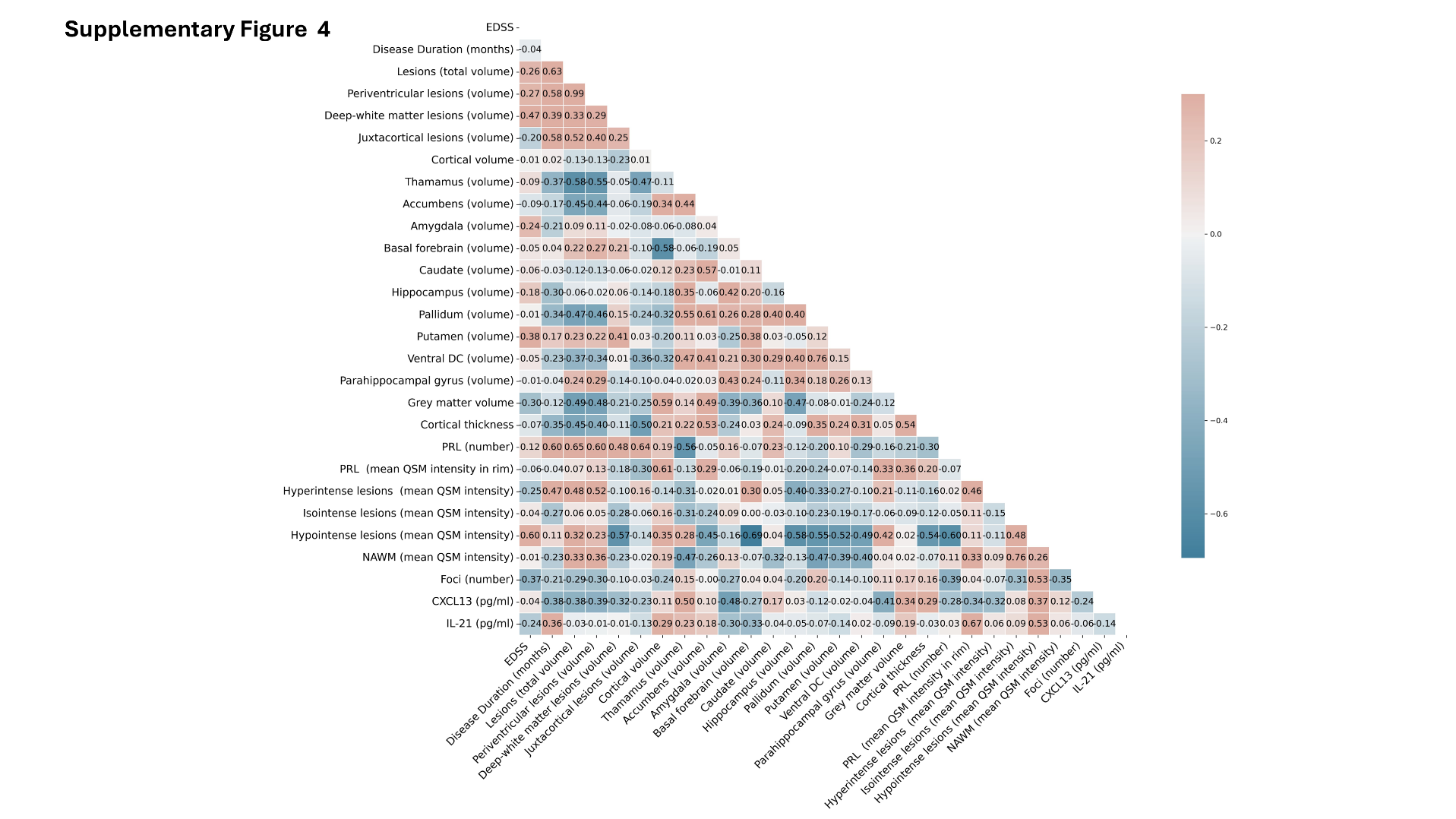


**Correlation matrix among demographic, clinical, and MRI parameters in MS patients**. Colors depict the type of relationship, with red showing positive and blue negative. The numbers inside the boxes represent the Spearman r of the respective correlations.
